# *UGT1A1* Variants c.864+5G>T and c.996+2_996+5del of a Crigler-Najjar Patient Induce Aberrant Splicing in Minigene Assays

**DOI:** 10.3389/fgene.2020.00169

**Published:** 2020-03-06

**Authors:** Linda Gailite, Alberto Valenzuela-Palomo, Lara Sanoguera-Miralles, Dmitrijs Rots, Madara Kreile, Eladio A. Velasco

**Affiliations:** ^1^Scientific Laboratory of Molecular Genetics, Riga Stradins University, Riga, Latvia; ^2^Splicing and Genetic Susceptibility to Cancer, Instituto de Biología y Genética Molecular (CSIC-UVa), Valladolid, Spain

**Keywords:** UGT1A1, unconjugated hyperbilirubinemia, Crigler-Najjar, aberrant splicing, minigene, splice site

## Abstract

A large fraction of DNA variants impairs pre-mRNA splicing in human hereditary disorders. Crigler-Najjar syndrome (CNS) is characterized by a severe unconjugated hyperbilirubinemia caused by variants in the *UGT1A1* gene. We previously reported one CNS-type II patient with two splice-site variants in trans (c.864+5G>T and c.996+2_996+5del). According to MaxEntScan, both disrupt their corresponding donor sites (c.864+5G>T: 6.99 → 2.28; c.996+2_996+5del: 5.96 → −11.02), so they were selected for subsequent functional tests. Given the unavailability of patient RNA, we constructed an *UGT1A1* splicing-reporter minigene with exons 1–4 to characterize the underlying splicing anomaly. The variant c.996+2_996+5del generated two aberrant transcripts, Δ(E2) (exon 2 skipping/64%) and ▼(E2q135) (intron retention of 135-nt/36%), which lead to the loss of 18 conserved amino-acids and the gain of 45 new ones of a critical functional domain, respectively. The c.864+5G>T variant mainly produced the aberrant transcript Δ(E1q141) (141-nt deletion/70.4%) and the full-length isoform (29.6%). Δ(E1q141) would provoke the loss of 47 amino-acids of the N-terminal domain that encodes for substrate specificity. Thus, the three anomalous transcripts are likely to inactivate *UGT1A1*. Moreover, this patient is also homozygous for the promoter variant A(TA)7TAA that decreases *UGT1A1* expression by 70%, so the full-length transcript produced by c.864+5G>T would be even more reduced (<9%), thus supporting the diagnosis of CNS-type II. Therefore, minigenes represent valuable tools for the functional and clinical classifications of genetic variants.

## Introduction

Unconjugated hyperbilirubinemia ranges in severity from no detectable symptoms (most Gilbert’s syndrome individuals) to severe bilirubin toxicity in Crigler-Najjar syndrome type II patients and fatal accumulation of bilirubin in Crigler-Najjar syndrome type I patients. The clinical outcome of a UGT1A1 deficiency relies on the residual activity of the *UGT1A1* gene (OMIM#606433, Gene ID: 54658). In Gilbert’s syndrome patients, UGT1A1 residual activity is ∼30% of the wild type activity, whereas in Crigler-Najjar type II and type I patients it is below 10 and 0%, respectively (Arias et al., 1969; Seppen et al., 1994; Kadakol et al., 2001; Maruo et al., 2016; Wagner et al., 2018).

We have previously detailed a patient with clinically diagnosed Crigler-Najjar syndrome type II caused by multiple allelic variants in the *UGT1A1* gene (Gailite et al., 2018). One of these variants was a novel deletion of four intronic nucleotides after exon 2 (NG_033238.1:g.11895_11898del, c.996+2_996+5del), potentially affecting the donor splice site. Another one was a nucleotide substitution in the +5 position of the first exon-intron splice junction (NG_033238.1:g.5884G>T, c.864+5G>T, rs777807265). Both variants were in trans and affected the 5′ splice sites; however, the absence of functional studies limited their clinical interpretations (Gailite et al., 2018). Interestingly, more than two decades ago, splice site variants were first proposed as a possible cause of unconjugated hyperbilirubinemia (Gantla et al., 1998).

Splicing is a critical gene expression step whereby introns are accurately removed from the precursor mRNA and exons are consecutively joined. Alternative splicing is a highly-regulated mechanism that is responsible for transcriptome and protein diversity. Indeed, it has been estimated that about 95% of human genes undergo alternative splicing (Pan et al., 2008; Wang et al., 2008). This process is strictly controlled by ribonucleoproteins, splicing factors, and a variety of *cis*-regulatory elements including the 5′ and 3′ splice sites, the polypyrimidine tract, the branch point, and splicing enhancers and silencers (Cartegni et al., 2002). All of these components are potential targets of pathogenic variants that can disrupt splicing and consequently lead to disease. In fact, defective splicing is one of the most common mechanisms of genetic diseases, being responsible for ∼10% of all genetic diseases (Wang and Cooper, 2007; Fraile-Bethencourt et al., 2019b; Jaganathan et al., 2019). Despite the implementation of novel algorithms and deep learning methods, prediction of the effect of variants located outside of the canonical splice sites remains limited. Further, even for canonical splice site variants, it is impossible to precisely predict transcript outcomes. Therefore, splicing functional assays are necessary to demonstrate the splicing impact of a particular DNA variant and to evaluate its pathogenicity (Richards et al., 2015; Jaganathan et al., 2019). RT-PCR of patient RNA samples is the most direct method to test splicing. However, the main limitation of this method is the requirement of tissue expressing the gene of interest, and unfortunately this type of tissue sample is not always available. Resultantly, splicing reporter minigenes have emerged as an alternative approach to test a variant from the splicing perspective (Tammaro et al., 2014). Remarkably, minigenes of the splicing vector pSAD have been successfully used to assay more than 300 variants of the breast cancer gene *BRCA2* (Acedo et al., 2015; Fraile-Bethencourt et al., 2017, 2019b).

In this study, we constructed an *ad hoc UGT1A1* minigene with exons 1 to 4 (mgUGT1A1_ex1-4) with the purpose of testing the intronic variants c.864+5G>T and c.996+2_996+5del from the donor sites of exons 1 and 2, respectively.

## Materials and Methods

### Case Description

The DNA of a patient with unconjugated hyperbilirubinemia was isolated from peripheral blood and the *UGT1A1* gene was sequenced as described previously (Gailite et al., 2018). Four different genetic variants were identified. Two of them were known and had been previously characterized – NG_033238.1:g.3664A>C (c.1352A>C, rs3755319) and NG_033238.1:g.4963_4964TA[7] (c.-53_-52insTA, A(TA)7TAA, UGT1A1^∗^28, rs8175347). The pathogenicity of the two other variants, which were found in trans (NG_033238.1:g.11895_11898del -c.996+2_996+5del- and NG_033238.1:g.5884G>T -c.864+5G>T, rs777807265) was evaluated according to the American College of Medical Genetics guidelines (Richards et al., 2015); both variants were classified as likely pathogenic. The patient’s phenotype and molecular findings confirmed Crigler-Najjar syndrome type II. Because *UGT1A1* gene expression occurs almost exclusively in the liver and gastrointestinal system and not in blood [GTEx: (GTEx Consortium et al., 2017)], we were unable to test the effect of the c.996+2_996+5del and c.864+5G>T variants on splicing in the patient due to the lack of non-blood tissue.

The study was carried out in accordance with the approved protocol of the local Ethics Review Committee. All participants (patient and parents) gave written informed consent in accordance with the Declaration of Helsinki.

### *In silico* Analysis

*In silico* analysis of the wild type and mutant sequences was performed by using the MaxEntScan algorithm (MES) (Yeo and Burge, 2004) that is integrated within the bioinformatics tool Human Splicing Finder version 3.1 (HSF)^1^ (Desmet et al., 2009). The default threshold value of MES within HSF is 3.0 except for variant analysis where all the resultant splice site scores are reported.

### Variant Nomenclature

Variants and transcripts were described according to the Human Genome Variation Society (HGVS) guidelines on the basis of the *UGT1A1* GenBank sequence NM_00463.2. For simplification, transcripts were annotated with a shortened code, as described previously (Fraile-Bethencourt et al., 2019a; Lopez-Perolio et al., 2019): Δ (skipping or loss of exonic sequences), ▼ (inclusion of intronic sequences), E (exon), p (acceptor shift), and q (donor shift). When necessary, the exact number of skipped or retained nucleotides is indicated.

### Construction of the Minigene mgUGT1A1_ex1-4

The construct mgUGT1A1_ex1-4 was assembled in three steps by overlapping extension PCR or classical restriction digestion/ligation cloning with three intermediate constructs: mgUGT1A1_EX2, mgUGT1A1_EX2-4, and mgUGT1A1_EX1-4. All the inserts were amplified from patient DNA with Phusion High Fidelity polymerase (Thermo Fisher Scientific, Waltham, MA, United States) and primers indicated in Table 1.

**TABLE 1:** Primers for insert amplifications.

Primers	Sequence 5′ → 3′
mgUGT1A1_ex2_Ins-FW	GGTGGCGGCCGCTCTAGAACTAGTGGATCCAAAACTAGCACATTACCTGGA
mgUGT1A1_ex2 Ins-RV	GACGGTATCGATAAGCTTGATATCGAATTCAAAATGATACTTCTGAGTGTGG
mgUGT1A1_ex3-4_*Eco*R1-FW	TATATAGAATTCCTCTAAGAGACTCAAAAGTGT
mgUGT1A1_ex3-4_*Hin*dIII-RV	TATATAAAGCTTAATGGGGGAAATAAAATTCTAAAT
mgUGT1A1_ex1_Ins-FW	AGTCACCTGGACAACCTCAAAGGCACCTTTCATTCAGATCACATGACCTTC
mgUGT1A1_ex1_Ins-RV	GATGCAAAATCCAGGTAATGTGCTAGTTTTAGCACACAGAGTAAAATGTCC

**FIGURE 1:**
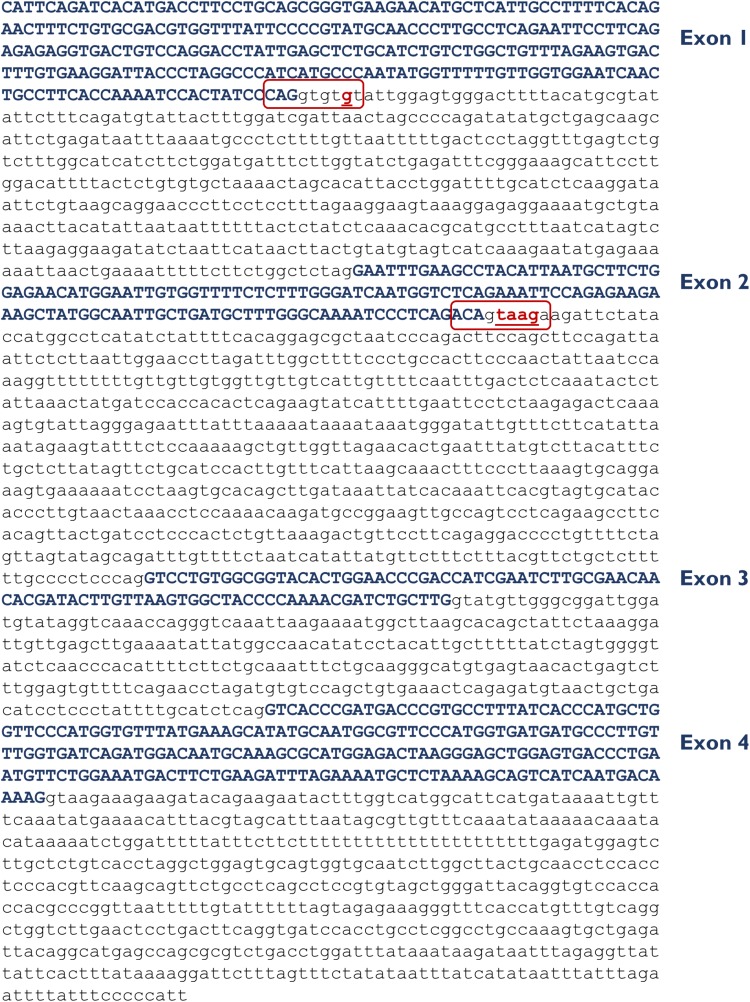
Insert sequence (2,717 bp) of minigene mgUGT1A1_ex1-4. Exons are indicated in uppercase and variants c.864+5G>T (intron 1) and c.996+2_996+5del (intron 2) are shown in red and underlined. The donor splice sites of exons 1 and 2 are boxed.

Mutant (c.996+2_996+5del) and wild type exon 2 from the patient were subcloned into the pSAD vector (Acedo et al., 2015) by overlapping extension PCR. Exons 3 and 4 were then inserted between the *Eco*RI and *Hin*dIII restriction sites of mgUGT1A1_ex2. Finally, mutant (c.864+5G>T) and wild type exon 1 from the patient were introduced by overlapping extension PCR, creating a chimeric exon V1-*UGT1A1* exon 1. This chimeric exon joins and keeps the open reading frames of V1 exon and *UGT1A1* exon 1 (codons 200 to 288): [V1]…LKGTF-[UGT1A1]_HSDHMTFLQ……NCLHQNPLSQ. The structure of the final insert was: ex1 (267 bp) – ivs1 (234 bp)//ivs1 (251 bp) – ex2 (132 bp) – ivs2 (234 bp)/*Eco*RI/ivs2 (449 bp) – ex3 (88 bp) – ivs3 (283 bp) – ex4 (220 bp) – ivs4 (553 bp) (Figure 1). Minigene mgUGT1A1_ex1-4 was sequenced to check the presence of the wild type or the mutant alleles. Therefore, three different minigenes were obtained: I) wild type; II) c.864+5G>T; and III) c.996+2_996+5del.

### Transfection of Eukaryotic Cells

Approximately 10^5^ MCF-7 cells were grown to 90% confluency in 0.5 ml of medium (Minimum Essential Medium Eagle, 10% Fetal Bovine Serum, 1% Non-Essential Amino Acid Solution, 2 mM). Cells were transfected with 1 μg of each construct and 2 μl of Lipofectamine LTX (Life Technologies, Carlsbad, CA, United States). To inhibit nonsense-mediated decay, a 4-hr incubation with cycloheximide 300 μg/ml (Sigma-Aldrich, St. Louis, MO, United States) was performed. RNA was purified with the GeneMATRIX Universal RNA Purification Kit (EURx, Gdańsk, Poland) including on-column DNase I digestion.

### RT-PCR

Retrotranscription was carried out with 400 ng of RNA and the RevertAid First Strand cDNA Synthesis Kit (Thermo Fisher Scientific) using the vector-specific primer RTPSPL3-RV (5′-TGAGGAGTGAATTGGTCGAA-3′). Samples were incubated at 42°C for 1 hr, followed by 5 min at 70°C. Then, 400 ng of cDNA (final volume of 50 μl) were amplified with SD6-PSPL3_RT-FW (5′-TCACCTGGACAACCTCAAAG-3′) and RTpSAD-RV (Patent P201231427) (full-length transcript 832 nt) using Platinum-Taq DNA polymerase (Thermo Fisher Scientific). Samples were denatured at 94°C for 2 min, followed by 35 cycles of 94°C/30 s, 60°C/30 s, and 72°C (1 min/kb), and a final extension step at 72°C for 5 min. RT-PCR products were run on 1% agarose gels and sequenced at the Macrogen facility in Madrid, Spain.

### Capillary Electrophoresis of Fluorescent RT-PCR

To quantify all transcripts, semiquantitative fluorescent RT-PCRs were undertaken in triplicate with primers SD6-PSPL3_RT-FW and RTpSAD-RV (FAM-labeled) and Platinum Taq DNA polymerase (Thermo Fisher Scientific) under standard conditions except that 26 cycles were herein applied (Fraile-Bethencourt et al., 2017, 2018). FAM-labeled products were run with LIZ-1200 Size Standard (Thermo Fisher Scientific) at the Macrogen facility and analyzed with Peak Scanner software V1.0 (Thermo Fisher Scientific). Only peak heights ≥ 50 RFU (Relative Fluorescence Units) were considered.

## Results

*In silico* analysis of the wild type and mutant sequences with MES revealed that variant c.864+5G>T severely affected the exon 1 donor site (6.99 → 2.28), while c.996+2_996+5del caused a complete disruption of the exon 2 donor site (5.96 → −11.02). Therefore, both variants were judged to have the potential to impair splicing and were consequently selected for subsequent functional analysis.

**FIGURE 2:**
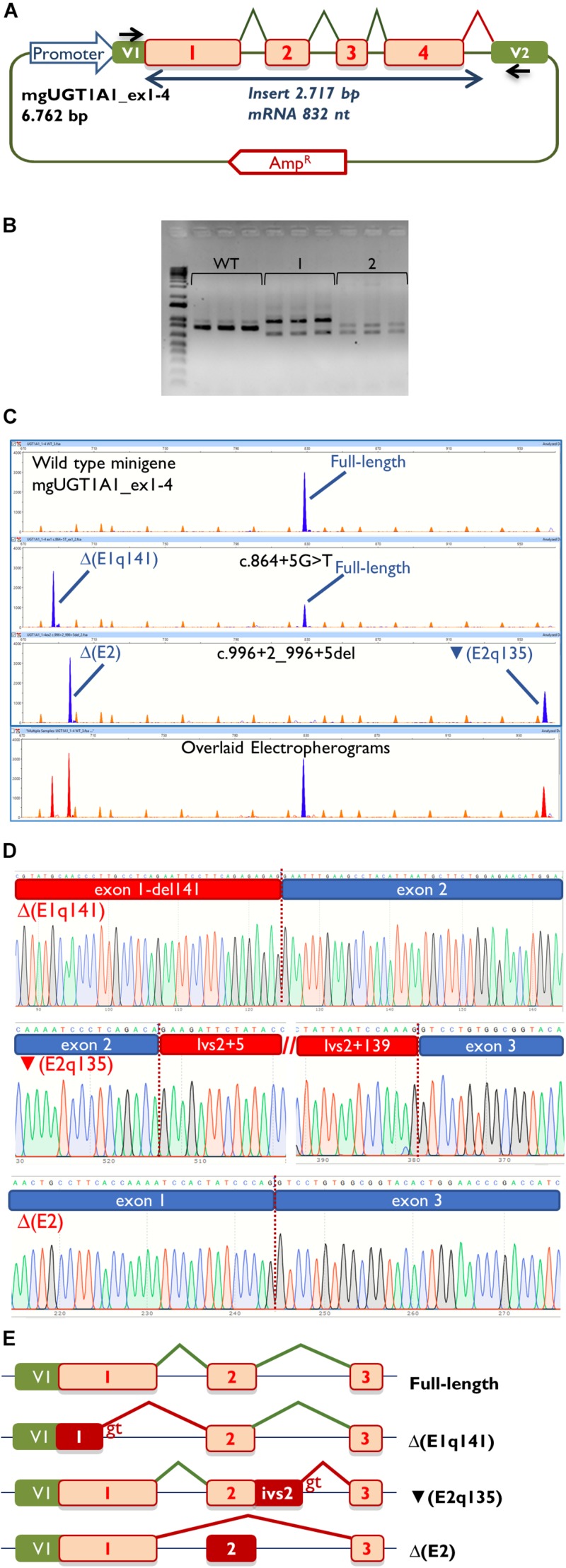
Splicing functional assays of minigene mgUGT1A1_ex1-4. **(A)** Structure of the construct. Splicing reactions in eukaryotic cells are represented by broken lines; specific vector exon primers are shown as black arrows in V1 and V2. **(B)** Agarose gel electrophoresis of RT-PCR products (in triplicate) of the wild type and mutant minigenes: 1, c.996+2_996+5del; 2, c.864+5G>T. The 1-kb DNA plus ladder is shown on the left. **(C)** Fragment analysis of FAM-RT-PCR products of the wild type and mutant minigenes experiments. Transcripts are shown as blue peaks while the LIZ1200 size standard is shown as orange peaks. Below is shown an electropherogram where the wild type and mutant ones have been overlaid. In this case aberrant transcripts are shown as red peaks. **(D)** Sequences of aberrant transcripts. **(E)** Diagrams of splicing events in the full-length and anomalous transcripts.

Then, the *UGT1A1* minigene with exons 1 to 4 was constructed from patient DNA in several steps as indicated in Materials and Methods. Therefore, we obtained three different minigenes: wild type, c.864+5G>T and c.996+2_996+5del. Remarkably, our construct contained a chimeric fusion of the vector reporter exon V1 and *UGT1A1* exon 1 that preserved its donor site (Figure 2A), so it was plausible to check variants affecting this site. Functional analysis of the wild type construct showed a stable transcript of the expected size (832 nt) and structure [V1 (28 nt)- ex1 (267 nt)- ex2 (132 nt)- ex3 (88 nt)- ex4 (220 nt)- V2 (97 nt)] (Figures 2B,C), so it was used for further testing.

**FIGURE 3:**
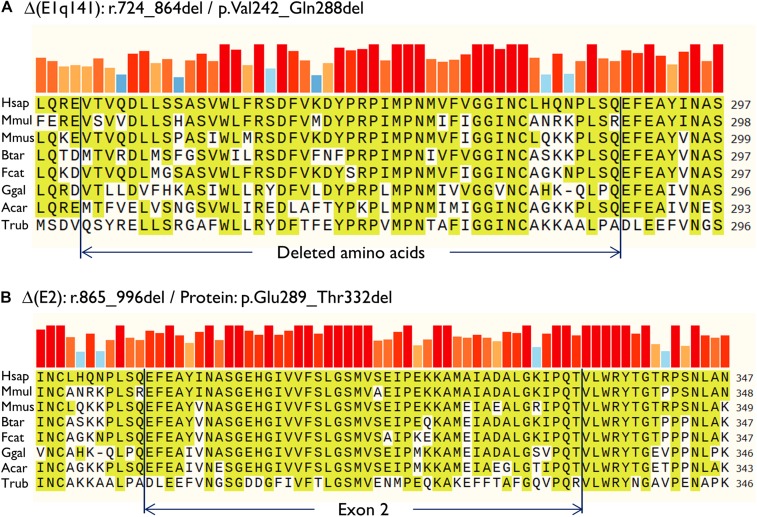
Amino acid conservation of the deleted sequences of the anomalous transcripts **(A)** Δ(E1q141) and **(B)** Δ(E2). Protein sequences were aligned with the Align tool of the Uniprot database (https://www.uniprot.org/align/). The alignment file was visualized with SnapGene Viewer version 4.3.11. The conserved residues are highlighted and the colored bars above the protein sequence show the degree of conservation where a red bar indicates the maximum level of conservation (100%). Organisms: Hsap, Homo sapiens; Mmul, macaque (*Macaca mulatta*); Mmus, mouse (*Mus musculus*); Btar, cow (*Bos Taurus*); Fcat, cat (*Felis catus*); Ggal, chicken (*Gallus gallus*); Acar, Anole lizard (*Anolis carolinensis*); Trub, fugu (*Takifugu rubripes*).

Then, both mutant minigenes were transfected and the RNA was purified and retrotranscribed. RNA assays of variant c.864+5G>T showed two transcripts (Figures 2B,C) that were subsequently sequenced (Figure 2D). The main transcript was the aberrant isoform Δ(E1q141) (70.4 ± 0.8%), generated using an alternative donor site of exon 1 (MES = 5.88), 141 nt upstream (Figures 2D,E). This variant also produced the full-length transcript in a reasonable proportion (29.6 ± 0.8%). It was surmised that Δ(E1q141) would keep the reading frame (r.724_864del) that would lead to the loss of 47 amino acids (p.Val242_Gln288del) of the N-terminal domain that encodes for substrate specificity of UGT1A1. The overall conservation of the UGT1A1 protein in vertebrates is 30.2% (161 out of 533 amino acids; Supplementary Figure S1). Particularly, 12 amino acids (25.5%) of the interval Val242_Gln288 are strictly conserved in vertebrates and 25 in mammals (Figure 3A), suggesting a relevant role in UGT1A1 function.

In contrast, c.996+2_996+5del produced two aberrant transcripts to substantial degrees: exon 2 skipping [Δ(E2)] (r.865_996del; 64.0 ± 0.4%) and a 135-nt insertion of intron 2 [▼(E2q135)] (r.996_997ins996+5_996+139; 36.0 ± 0.4%) by alternative use of a cryptic intronic 5′ splice site (MES = 3.43). It was established that both transcripts, Δ(E2) and ▼(E2q135), would keep the reading frame [p.Glu289_Thr332del and p.Thr332_Val333ins(45), respectively]. Whereas Δ(E2) was predicted to delete 44 amino acids, ▼(E2q135) was speculated to insert 45 new amino acids with no similarities with any known human protein. In both cases, the resultant impact on protein function remains to be established. In any case, 18 out of the 44 deleted residues of exon 2 are conserved in vertebrates (36 in mammals), suggesting that they are essential for an adequate protein function (Figure 3B).

## Discussion

We previously identified two putative variants that affected the canonical donor splice sites of *UGT1A1* exons 1 and 2 (Gailite et al., 2018). Both variants were predicted by MES to disrupt their respective splice site. Therefore, splicing assays were highly recommended to prove their impact on splicing. The *UGT1A1* gene is almost exclusively expressed in liver cells; however, patient RNA was not available. Alternatively, splicing reporter minigenes constitute a valuable tool for the characterization of splicing outcomes and have been judged as comparable with expression studies performed using patient liver cells (Gantla et al., 1998).

The *UGT1A1* gene generates several protein isoforms where the amino acid sequence from exon 1 is responsible for substrate specificity (e.g., bilirubin binding) and exons 2–5 are shared with other UGT1A isoforms (Girard et al., 2007). Additionally, *UGT1A1* isoforms can undergo alternative splicing by using an alternative exon 5, accounting for 27 different mature mRNA species (Jones et al., 2012). UGT1A1 transforms lipophilic molecules (mainly unconjugated bilirubin) into soluble metabolites, enabling them to be excreted from the body. Decreased or zero activity of the protein leads to the accumulation of bilirubin, resulting in unconjugated hyperbilirubinemia syndromes such as Crigler-Najjar syndrome type II.

Therefore, we designed and constructed a *UGT1A1* minigene with exons 1–4 as both variants had the potential to affect the splicing of exons 1 and 2 but not exon 5. Remarkably, the main limitations of minigenes are the first and last exons of any gene because the splicing vector (pSAD) contains a reporter vector exon V1 which launches the splicing reaction and a vector exon V2 which finishes it (Figure 2A). We had previously constructed a minigene with a fusion of the last exon of the *BRCA2* gene (exon 27) and *V2* (Acedo et al., 2015). Here, we report a chimeric V1-exon 1 that was generated by overlapping extension PCR. Moreover, this construct was fully functional and induced a full-length transcript of the expected size and sequence (Figures 2B,C). Likewise, the mutant constructs (c.864+5G>T and c.996+2_996+5del) yielded clean splicing patterns (Figure 2C), where three aberrant transcripts [Δ(E1q141), Δ(E2), and ▼(E2q135)] were identified with high resolution and sensitivity by fluorescent fragment analysis (Fraile-Bethencourt et al., 2019a). Thus, c.864+5G>T induced a deletion of 141 nt of exon 1, similar to previously reported variant c.864+1G>C (Gantla et al., 1998), but also a significant proportion of the full-length transcript (29.6%) (Figures 2B–E). Although in some cases splicing does not differ among different cell lines (Sanz et al., 2010; Sharma et al., 2014), these splicing outcomes have been obtained in MCF-7 cells (human breast adenocarcinoma) and should be further confirmed in liver cell lines.

Analysis of the aberrant transcripts suggested that active protein would not be formed. Δ(E1q141) was found to entail the deletion of 47 amino acids of the UGT1A1 N-terminal domain that encodes for substrate specificity (exon 1, 288 amino acids) (Kadakol et al., 2000). Hence, protein functionality would likely be altered. In contrast, the splicing tests of c.996+2_996+5del revealed two abnormal transcripts [Δ(E2) and ▼(E2q135)] (Figures 2B–E). Both RNA isoforms were found to keep the reading frame. The common exons 2 to 5 of the *UGT1A1* genes encode for the domain that binds the donor substrate UDP-glucuronic acid (Kadakol et al., 2000). Moreover, exon 2 skipping [Δ(E2)] was predicted to result in the loss of 18 amino acids conserved in vertebrates (Figure 3). Consequently, it is plausible that either the loss of exon 2 codons or the insertion of 45 new amino acids [▼(E2q135)] would impair UGT1A1 function; however, our interpretation of these in-frame transcripts should be approached with a degree of caution. Interestingly, a missense variant at the conserved Gly 308 (p.Gly308Glu) has been reported as pathogenic at the UGT1A1 mutation database^2^, thus supporting the relevance of this protein region.

For Crigler-Najjar syndrome type II, the functional activity of UGT1A1 is below 10%. Our Crigler-Najjar syndrome type II patient with 29.6% expression of the canonical transcript from one allele (c.864+5G>T) does not appear to fulfill this criterion. Also family segregation data from our previous study suggested that each of the variants separately did not reduce UGT1A1 enzyme activity enough to cause Crigler-Najjar syndrome type II (Gailite et al., 2018).

However, there are two possible explanations as to why *in vivo* expression could be even more reduced. First, this patient is homozygous for the *UGT1A1* promoter A(TA)7TAA variant which leads to 70% reduction in UGT1A1 expression. This may affect and strengthen the deleterious effect of variants located in the coding part of the gene (Kadakol et al., 2001). Secondly, homo- and heterodimerization of isoforms may have an impact on UGT1A1 activity, where the amount of wild type protein is reduced by dimerization with aberrant isoforms (Ghosh et al., 2001). This dominant-negative effect would account for some Crigler-Najjar syndrome type II patients with only one pathogenic allelic variant (Suzuki et al., 2014). Yet, this effect was not observed in the parents of the patient.

According to the American College of Medical Genetics guidelines (Richards et al., 2015), our functional studies now allow both variants to be classified as pathogenic as they fulfill the following criteria: **PS3** (well-established *in vitro* or *in vivo* functional studies supportive of a damaging effect on the gene or gene product), **PM2** (absent from controls (or at extremely low frequency if recessive) in Exome Sequencing Project, 1000 Genomes, or ExAC), **PM3** (for recessive disorders, detected in *trans* with a pathogenic variant), **PP3** [multiple lines of computational evidence support a deleterious effect on the gene or gene product (conservation, evolutionary, splicing impact, etc.)], and **PP4** (patient’s phenotype or family history is highly specific for a disease with a single genetic etiology).

## Conclusion

In conclusion, we have shown that functional assays utilizing minigenes are very useful for checking the splicing impacts of potential spliceogenic DNA variants. Such assays are absolutely essential for the clinical interpretation of this type of variants. Indeed, they allowed the molecular confirmation of Crigler-Najjar syndrome type II diagnosis in our patient. Furthermore, although the splicing vector pSAD was initially developed to study the breast cancer genes *BRCA1* and *BRCA2* (Acedo et al., 2015; Fraile-Bethencourt et al., 2017), it has also been demonstrated to be a powerful tool to test other disease genes such as *SERPINA1* (Lara et al., 2014), *CHD7* (Villate et al., 2018), and *UGT1A1*, as well as others currently under investigation^3^.

## Data Availability Statement

All datasets generated for this study are included in the article/Supplementary Material.

## Author Contributions

MK proposed the concept of the study and involved in the patient’s clinical characterization. MK, DR, and LG performed the patient’s genetic analysis and the family segregation analysis. LG and EV designed the functional study. AV-P, LS-M, and EV involved in construction of the minigenes and all steps of the functional study. EV and LG prepared the manuscript. All authors critically reviewed the manuscript.

## Conflict of Interest

The authors declare that the research was conducted in the absence of any commercial or financial relationships that could be construed as a potential conflict of interest.

## Footnotes

1 http://www.umd.be/HSF3/

2 https://databases.lovd.nl/shared/genes/UGT1A1
