# Supplementary material for: UGT1A1 Variants c.864+5G>T and c.996+2_996+5del of a Crigler-Najjar Patient Induce Aberrant Splicing in Minigene Assays
Source: Front Genet. 2020 Mar 6;11:169. doi: 10.3389/fgene.2020.00169 (PMC7067894; doi:10.3389/fgene.2020.00169)
Supplement: Supplementary file 1 [file Data_Sheet_1.PDF]

Aligned using an external algorithm

Consensus

1. SP|P22309|UD11_HUMAN
2. TR|F7GW14|F7GW14_MACMU
3. SP|Q63886|UD11_MOUSE
4. TR|A7YWD3|A7YWD3_BOVIN
5. TR|M3WT94|M3WT94_FELCA
6. TR|F1P1M1|F1P1M1_CHICK
7. TR|G1KUU6|G1KUU6_ANOCA
8. TR|H2T5F6|H2T5F6_TAKRU

```
   MAxxxxxxx-xxLxxxLLLCxxxPxxxxxGKLLVxPxDGSHWLSMxxxxxxLxQxGHxxVVxAPEASxxIK-EExFYTLK
1  MAVESQGGR--PLVLGLLLCVLGPVVSHAGKILLIPVDGSHWLSMLGAIQQLQQRGHEIVVLAPDASLYIR-DGAFYTLK  77
2  MATGLQVP-LPQLATGLLLLLSVQPWAESGKVLVVPTDGSHWLSMREAVRELHARGHQAVVLTPEVKMHIK-EENFFTLT  78
3  MTVVCWSSRLLLLLPYLLLCVFGPSASHAGRLLVFPMDGSHWLSMLGVIQQLQQKGHEVVVIAPEASIHIK-EGSFYTLR  79
4  MTAGSQGDR--PVILLLLLCALGPSVSQGGKLLVVPVDGSHWLSLVGPLQPLQQKGHDIVVLAPDASIYIK-EEAFYTLK  77
5  MAARSRGPR--PLVLSLLLCALNPLLSQGGKLLLVPMDGSHWLSLFGVIQRLHQRGHDVVVVAPEASVYIK-EGAFYTLK  77
6  MALVLPSH--PQVSVSLLLLLSVLSLAAGGKLLVVSVDGSPWFSVLEMLEVLKQKGHEIVVVAPEASLNVKPSESF-ILK  77
7  MA-----P--VFYSNVILLLLASLNLASGGKLLVIPMDGSHWLSMKPVLKQLRQNGHEMVIIAPEVSIHIKPEEDFYTLK  73
8  MRGS---GWFPTLGLLAWFCCFGPRPVEAGKVLVLPVDGSHWLSMKILVKELVQRGHDVLVLVPETSLLIKSSEN-YRTE  76

   xYPVPFxxExxxxxxxxLxxxxFExxxFLxRxxxxxxxxKxxSAxxLxxCxHLLxNxELMxxLxESxFDAxLTDPFLPCG
1  TYPVPFQREDVKESFVSLGHNVFENDSFLQRVIKTYKKIKKDSAMLLSGCSHLLHNKELMASLAESSFDVMLTDPFLPCS  157
2  TYAIPWTQDEFDRLVLGHTQWFFETEHLLKRYSRSMAIMNNMSLVFHRSCVELLHNEALIRHLNATSFDVVLTDPVNLCG  158
3  KFPVPFQKENVTATLVELGRTAFNQDSFLLRVVKIYMKVKRDSSMLLAGCSHLLHNAEFMASLEESHFDALLTDPFLPCG  159
4  RYPVPFRREDLEETFISLGRTVFEDDPFLKRVIKTYQKIKKDSALLLSACSHLLHNKELMASLTASSFDAVLTDPFLPCG  157
5  SYPVPFRREDVEASFTGLGLGVFEKKPFLQRVVATYKRVKKDSALLLSACSHLLYNEELMASLAESGFDAMLTDPFLPCG  157
6  TYPASSKQKGMAGNLQLFLQEALEEGSFLGRFFRVRENMKNLSASAVIDCENLLYNKELIKYLEESKFDALLTDPILSCG  157
7  TYPVPFTSEEMNENIQSFSDHVFEDVPFLVMIAKTFELMKKSSAMFLGSCTHLLYNEELKMYFRENKFDAVFSDPFWPCG  153
8  IYQVPYSKEDLDGNFNELKDGLFDKPPTMADIFINVERLVTFTTMQVSGCESLLRNQPLMTRLQEQGFEVVLTDPFLPCG  156

   xIVAxYLxxPxVFFLxxLPCxLDFxATQCPxPPSYVPRxLSxNSDHMTFLQRVKNMLxxxSxxFLCxxVYxPYxxLASEx
1  PIVAQYLSLPTVFFLHALPCSLEFEATQCPNPFSYVPRPLSSHSDHMTFLQRVKNMLIAFSQNFLCDVVYSPYATLASEF  237
2  AVLAKYLSIPTVFFLRNIPCDLDFKGTQCPNPYSYIPKLLTTNSDHMTFLQRVKNMLYPLALSYICHAVSAPYASLASEL  238
3  SIVAQYLTVPTVYFLNKLPCSLDSEATQCPVPLSYVPKSLSFNSDRMNFLQRVKNVLLAVSENFMCRVVYSPYGSLATEI  239
4  PIVAQYLSVPAVFFLNGLPCSLDFQGTQSPSPPSYVPRYLSFNSDHMTFLQRVKNMFITLSESLLCDMVYSPYGLLASEI  237
5  PIVALRLALPVVFFLNSLPCGLDFQGTRCPSPPSYVPRVLSLNSDHMTFLQRVKNMLILGSEGFLCNVVYSPYASLASEV  237
6  AILAEYLSIPSVYFMRLIPCGFDSEASQCPSPPSYIPRAFSDLTDHMNFLQRVKNVIFDTSNLFLCDFLFKPYDKLASEF  237
7  QIVAEYLEIPSVFFLRGIPCGYEFEATQCPRPPSYVPRGFTRYSDHMTFPQRVKNMLFHFTEFFLCSSVYTPYAKLASEF  233
8  SILSHLFNVPAVYFLRGLPCELDLKANQCPAPPSYVPMAFSGNSDVMNFPQRVKNMLMYFVQSYMCKIIYREFDRLVTRH  236

   LQxxVTVxDLxSxASVWLxRSDFVxDYPRPIMPNMxFIGGINCAxKxPLSQEFEAYVNASGEHGIVVFSLGSMVSEIPEK
1  LQREVTVQDLLSSASVWLFRSDFVKDYPRPIMPNMVFVGGINCLHQNPLSQEFEAYINASGEHGIVVFSLGSMVSEIPEK  317
2  FEREVSVVDLLSHASVWLFRSDFVMDYPRPIMPNMIFIGGINCANRKPLSREFEAYINASGEHGIVVFSLGSMVAEIPEK  318
3  LQKEVTVQDLLSPASIWLMRSDFVKDYPRPIMPNMVFIGGINCLQKKPLSQEFEAYVNASGEHGIVVFSLGSMVSEIPEK  319
4  LQTDMTVRDLMSFGSVWILRSDFVFNFPRPIMPNIVFVGGINCASKKPLSQEFEAYVNASGEHGIVVFSLGSMVSEIPEQ  317
5  LQKDVTVQDLMGSASVWLFRSDFVKDYSRPIMPNMVFIGGINCAGKNPLSQEFEAYVNASGEHGIVVFSLGSMVSAIPKE  317
6  LQRDVTLLDVFHKASIWLLRYDFVLDYPRPLMPNMIVVGGVNCAHK-QLPQEFEAIVNASGEHGIVVFSLGSMVSEIPMK  316
7  LQREMTFVELVSNGSVWLIREDLAFTYPKPLMPNMIMIGGINCAGKKPLSQEFEAIVNESGEHGIVVFSLGSMVSEIPMK  313
8  MSDVQSYRELLSRGAFWLLRYDFTFEYPRPVMPNTAFIGGINCAKKAALPADLEEFVNGSGDDGFIVFTLGSMVENMPEQ  316

   KAMEIADALGxIPQTVLWRYTGTxPxNLAKNTxLVKWLPQNDLLGHPKTRAFITHxGSHGIYEGICNGVPMVMMPLFGDQ
1  KAMAIADALGKIPQTVLWRYTGTRPSNLANNTILVKWLPQNDLLGHPMTRAFITHAGSHGVYESICNGVPMVMMPLFGDQ  397
2  KAMAIADALGKIPQTVLWRYTGTPPSNLANNTILVKWLPQNDLLGHPMTRAFITHAGSHGIYEGICNGVPMVMMPLFGDQ  398
3  KAMEIAEALGRIPQTVLWRYTGTRPSNLAKNTILVKWLPQNDLLGHPKTRAFITHSGSHGIYEGICNGVPMVMMPLFGDQ  399
4  KAMEIADALGKIPQTVLWRYTGTPPPNLAKNTKLVKWLPQNDLLGHPKTRAFITHSGSHGIYEGICNGVPMVMMPLFGDQ  397
5  KAMEIADALGKIPQTVLWRYTGTPPPNLAKNTILVKWLPQNDLLGHPKARAFITHSGSHGIYEGICNGVPMVMLPLFGDQ  397
6  KAMEIADALGSVPQTVLWRYTGEVPPNLPKNVKLVKWLPQNDLLAHPKTRAFITHGGSHGVYEGICNAVPMVLMPLFGDQ  396
7  KAMEIAEGLGTIPQTVLWRYTGETPPNLAKNTKLVKWLPQNDLLAHPKTRAFITHAGSHGVYEGICNGVPMVLMPLFGDQ  393
8  KAKEFFTAFGQVPQRVLWRYNGAVPENAPKNVKVMKWLPQNDLLAHPKAKVFITHGGTHGIYEGICNGVPMLMFPLFGDQ  396
```

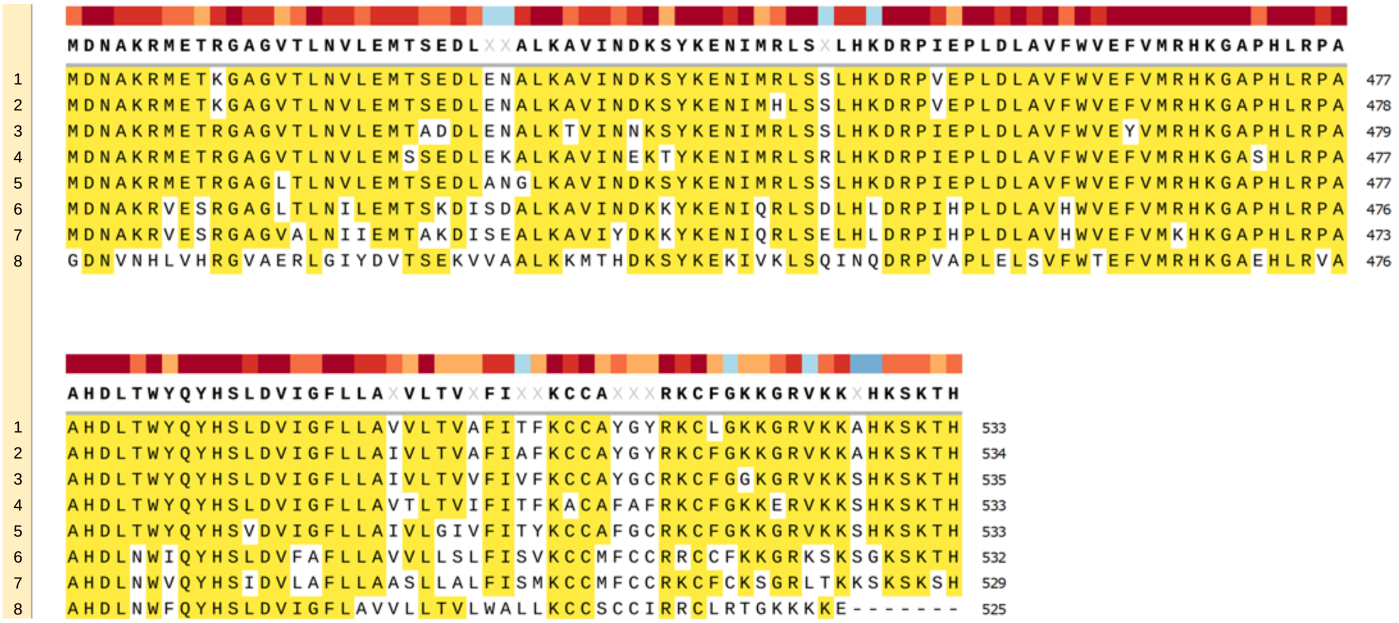

Supplementary Figure S1. Amino acid conservation of the UGT1A1 protein. Protein sequences were aligned with the Align tool of the Uniprot database (https://www.uniprot.org/align/). The alignment file was visualized with SnapGene Viewer version 4.3.11. The conserved residues are highlighted and the coloured bars above the protein sequence show the degree of conservation where a red bar indicates the maximum level of conservation (100%). Organisms: Hsap, Homo sapiens; Mmul, macaque (Macaca mulatta); Mmus, mouse (Mus musculus); Btar, cow (Bos Taurus); Fcat, cat (Felis catus); Ggal, chicken (Gallus gallus); Acar, Anole lizard (Anolis carolinensis); Trub, fugu (Takifugu rubripes).

Consensus Threshold: >50%

Compare to: the consensus

Amino acids that match the reference are marked with yellow highlighting.

Created: viernes, 10 de ene. de 2020

Last Modified: viernes, 10 de ene. de 2020
